# The Dead Emperor

**Published:** 1888-07-21

**Authors:** 


### The Dead Emperor.

Justice knows no distinction of race. Science takes no account of patriotic feeling. Every man owes a duty to his family, to his country, and to himself; but a still higher duty is owed to truth and right. The persons concerned, the issues at stake, and the interests involved in the recent events which have transpired in Germany have all been of a kind to make men ashamed of vainglorious motives and self-seeking aims, and to raise them to a high level of sober and dignified judgment. By the bedside of those who are mortally sick, and in the presence of the dead, even a frivolous person feels that self-repression is demanded. Much more, when the fate of empires as well as of individuals is trembling in the balance, will the man of culture, sense, and right feeling hold himself bound to maintain that just dignity of behaviour which alone befits the occasion. From a man of firm character and sterling worth the feeling will not pass away when he leaves the death chamber, but will remain as a controlling influence over his conduct for future time. Some such behaviour as this the world had a right to expect from all, including even the very servants of the household, who were privileged to share the anxieties and griefs of the late Emperor Frederick and his family. How much more was such conduct imperative on those to whose hands the most exalted science of the time was entrusted, and with whom lay the dread authority of pronouncing the words of life or death.

Great occasions reveal great men. How often also they shed a light on mean men. Nothing can make a man of little soul great or dignified; not all the rank, nor all the science, nor all the professional skill which the whole world can boast. If he be selfish, if he be vainglorious, if he be at heart mean and poor, let but the day come and the circumstance, and all the world will see that contempt and not honour is his just reward.

We had hoped the time was past when it was possible for the highest authorities of the noble and humane profession of medicine to afford to the world a spectacle of wrangling and heartburning of which even tavern brawlers might be ashamed. It was known, or at least believed, that during the Emperor Frederick's life there were differences of judgment of a serious character, not only concerning the nature of the malady, but also about the general principles, as well as the minor details of treatment. But those differences, which to the outside world appeared so great and striking when stated in popular language, did not affect the medical mind in the same way. From a very early stage in the history of the late Emperor's illness, every competent medical man was convinced that death was imminent—that it was a case of weeks, or at most of months. Under such circumstances there were but two courses possible: a radical operation, or the most careful general management without such an operation. What were the prospects of an operation? They were of a palliative—but not of a curative—character. What, on the other hand, were the risks? So great and so unavoidable, that even in the case of a young and robust person the chances of death would have been as twenty to one; whilst in the case of a patient of the Emperor's age and small recuperative power, those chances were probably as a thousand to one. The most that could be said for a radical operation was that it might possibly prolong life for a few more months, whilst it would almost certainly not prolong it at all, but bring on a fatal climax immediately.

That was the situation in the early part of Sir Morell Mackenzie's attendance on the case. Is it not obvious that what was required was a plain statement of the facts, and a clear explanation of the possibilities, in order that the Emperor and his family, and other responsible persons, might themselves make the decision on which such great issues depended? When such a decision was once made, any medical specialist of adequate experience was perfectly competent to carry out the details of treatment. Of course if ten such specialists were entrusted with a case of the kind it is not only probable, but certain, that each would manage the details in his own way, and no two of them would manage them in an exactly identical way. Equally, of course, they would all conduct the case on similar, although not on identical lines.

The Emperor's family, and others who had a right to share the responsibility of decision, elected to pursue the course which seemed to Sir Morell Mackenzie's ripe judgment the best; and when once such a decision was arrived at the critical period of scientific judgment was past. All differences should then have been loyally sunk and forgotten; every medical man connected with the case should have considered it his highest duty to contribute such suggestions of detail as his own peculiar experience had furnished him with. Each should have felt bound in honour to show to the Emperor's family and the world the practical agreement of scientific opinion, and the dignified magnanimity which culture, good feeling, and knowledge of the world ought always to produce.

Instead of this, what have we seen? Men snarling like wolves behind the bars of their cage, and only kept from breaking out into uncontrollable violence by the whip of public opinion, an opinion which would have heaped upon them an overwhelming scorn if they had not had the self-control to await the patient Emperor's death. No sooner is he dead and buried than the rage bursts forth. Everyone is anxious to show that his knowledge was the greatest, his opinion the soundest, his advice the best. The physicians not in charge were philosophers and men of genius, and if their judgment had been followed the patient would have been alive and well at this hour. The physicians in actual authority were quacks and charlatans, who deceived their patient, imposed upon the public, and brought about one of the greatest calamities which could have visited a great empire. Such is the inference which we are covertly invited to draw from the medical report published under the authority of responsible German physicians. The public must form their own conclusions. Every thoughtful man, both in Germany and England, will at least understand that one side only of the case has been published. The other side is bound in fairness to be heard before any decision is arrived at. It is understood that a command has been issued for the preparation of a statement by those who had responsible charge of the case. When such a statement is published, the means will be available for the forming of right conclusions. In the meantime the world will wait. But, so far as events have as yet transpired, there has been presented to the civilised peoples of the nineteenth century the spectacle of men of the highest scientific attainments and of the most dignified professional position behaving towards other professional men of equal eminence in a manner of which the best that can be said is that it is a scandal and a disgrace to the whole scientific world.

